# Translation, Cross-Cultural Adaptation, and Validation of the Hospital Consumer Assessment of Healthcare Providers and Systems (HCAHPS) into the Malay Language

**DOI:** 10.3390/ijerph16112054

**Published:** 2019-06-10

**Authors:** Ahmad Badruridzwanullah Zun, Mohd Ismail Ibrahim, Ariffin Marzuki Mokhtar, Ahmad Sukari Halim, Wan Nor Arifin Wan Mansor

**Affiliations:** 1 Department of Community Medicine, Universiti Sains Malaysia, Kota Bharu 16150, Kelantan, Malaysia; drahmadbadrur987@gmail.com; 2 Hospital Universiti Sains Malaysia Management Unit, Kubang Kerian, Kota Bharu 16150, Kelantan, Malaysia; ariffinm@usm.my (A.M.M.); ashalim@usm.my (A.S.H.); 3 Unit of Biostatistics and Research Methodology, School of Medical Sciences, Universiti Sains Malaysia, Kubang Kerian, Kota Bharu 16150, Kelantan, Malaysia; wnarifin@usm.my

**Keywords:** HCAHPS, patient perception, translation, validation, Hospital USM

## Abstract

Background: Patient feedback is an important tool in assessing health system quality. The Hospital Consumer Assessment of Healthcare Providers and Systems (HCAHPS) was developed in 2006 as a standardized instrument to assess patient perceptions in the United States of America. This study aimed to translate and validate the HCAHPS questionnaire into the Malay language in order to assess patient perceptions of health services in Malaysia. Methods: The original HCAPHS in English was translated into Malay based on the established guideline. The content validation involved an expert panel of 10 members, including patients. The face validation pilot testing of the HCAHPS-Malay version was conducted among 10 discharged patients. The exploratory factor analysis (EFA) used principal axis factor, and varimax rotation was established based on a cross-sectional study conducted among 200 discharged patients from Hospital Universiti Sains Malaysia (Hospital USM). Results: The overall content validity index was 0.87, and the universal face validity index was 0.82. From the EFA, the factor loading value ranged from 0.652 to 0.961 within nine domains. The internal consistency reliability with Cronbach’s alpha was 0.844. Conclusion: The HCAHPS-Malay is a reliable and valid tool to determine patients’ perception of healthcare services among inpatients in Hospital USM based on the content and face validation result together with a good construct validity and excellent absolute reliability. Further testing on HCAHPS-Malay version in other settings in Malaysia needs to be done for cross-validation.

## 1. Introduction

In today’s competitive market, it is crucial for the healthcare system to provide a high quality of care. Research has shown that patients with a positive perception of healthcare services have better clinical outcomes [1,2]. In addition, satisfaction levels can also influence patients’ utilization of healthcare services, as satisfied patients tend to take an active role in their own healthcare management [3]. Worldwide, patients’ feedback has become an important tool in the process of monitoring and assessing the quality of health systems, leading to the implementation of improvement strategies [4,5].

The feedback from the patient can be in the form of their perceptions, experience, or satisfaction level. Several survey instruments have been developed to assess both forms of patient feedback, either satisfaction or perception [6,7,8,9]. Most instruments are specific to a country’s health system or type of healthcare facility. There are a limited number of survey instruments used as a standardized instrument for a national survey program. One from the United States is the Hospital Consumer Assessment of Healthcare Providers and Systems (HCAHPS) survey, which is one of the most widely used questionnaires to assess patient perception/satisfaction levels, because it provides data for benchmarking and makes comparison across healthcare institutions possible.

The HCAHPS survey was developed by the Centers for Medicare and Medicaid Services in partnership with the Agency for Healthcare Research and Quality in 2006 [10]. Since then, this instrument has become a standardized survey instrument to measure the quality of health services by assessing patients’ perceptions of and satisfaction with hospital care in the United States of America (USA). The HCAHPS survey’s findings have subsequently been used as a reimbursement and incentive mechanism for provider improvement [11]. Therefore, US hospitals try to achieve higher HCAHPS scores in order to receive more funding by improving the patient experience [12]. Moreover, the HCAHPS findings are posted for public review, helping consumers in their decision-making process by enabling comparisons across hospitals [10,12]. The HCAHPS questionnaire has been translated and used in various countries, including Canada, China, The Netherlands, France, Finland, Germany, Italy, Greece, Poland, and Arab countries [12,13,14,15,16]. The HCAHPS survey was selected for the European Commission RN4CAST project, which involved 12 countries (England, Belgium, Germany, Finland, Ireland, Greece, Norway, The Netherlands, Sweden, Poland, Spain, and Switzerland) because of its potential to provide meaningful comparisons across healthcare systems between the participating European countries and the USA on established patient experience domains [15]. In Greece, the HCAHPS survey was used nationwide by the Ministry of Health to determine inpatient satisfaction in public hospitals after the implementation of health system reform following its financial crisis [9].

The Malaysian Ministry of Health has a policy on quality improvement in which one of the components is a patient perception/satisfaction survey. The national patient satisfaction survey for government hospitals was launched in 2011 [17] using a standardized instrument based on the SERVQUAL concept proposed by Parasuraman et al. [18]. Besides hospitals under the Ministry of Health, there are a few teaching hospitals under the Ministry of Education that provide healthcare services. One of the teaching hospitals is Hospital Universiti Sains Malaysia (Hospital USM), which is located in Kota Bharu, the capital city for Kelantan State. Hospital USM is one of the centers of excellence in the medical field and has 20 specialties and subspecialties with 816 beds in 39 wards, being a referral center for the East Coast of Peninsular Malaysia. Private hospitals and teaching hospitals have their own patient satisfaction surveys specific to their setting. Hospital USM is adopting a quality improvement initiative that includes a patient perception/satisfaction survey. Previously, Hospital USM used their own survey instrument to assess patient perception/satisfaction levels, but this hindered service quality comparisons with other healthcare facilities.

The objective of this study was to conduct the translation and adaptation process of the HCAHPS into the Malay language and to determine the validity and reliability of the translated HCAHPS Malay version. The literature search showed that there was a gap in the use of HCAHPS in the Malay language and in Malaysia generally, which indicates this study’s significance and valuable contribution.

## 2. Materials and Methods

A cross-sectional validation study was carried out from January to February 2019 in the Hospital USM, Malaysia. This study was conducted among 200 discharged patients selected from three wards: medical, surgical, and obstetrics and gynecology. The self-administered HCAHPS questionnaire was distributed to the selected patients with the aim of measuring their perceptions on the quality of care during their hospitalization.

### 2.1. HCAHPS Questionnaire

Hospital USM is a center of excellence in the medical field that aims to achieve international standards and recognition. The HCAHPS questionnaire was chosen to obtain health services quality measurement comparable with a developed country such as the USA. Based on its development and validation process, and taking into consideration its psychometric component, the HCAPHS questionnaire is a good, valid instrument to assess patient perceptions [12,19]. The HCAHPS needed to be translated into the Malay language before use in Malaysia. The Malay language is a national language in Malaysia, and most Malaysians can understand and communicate in the Malay language. Moreover, this study was conducted in Kelantan State where 99% of the population are ethnic Malay [20]. Having a valid Malay questionnaire version would help the respondents to give their actual experiences and opinions.

The HCAHPS questionnaire is composed of 22 items, with 18 items on the critical aspect of hospital experience and 4 screening items. The 18 items consist of 16 questions related to the recent hospital experience and 2 global ratings of care. The components on the hospital experience are communication with nurse (Q1–Q3), communication with doctor (Q5–Q7), responsiveness of hospital staff (Q4, Q11), the hospital environment (Q8, Q9), pain management (Q13, Q14), communication about medicine (Q16, Q17), and discharge information (Q19, Q20). The two global rating items are the overall rating of the hospital (Q21) and the willingness to recommend hospital to friends and family (Q22). The four screening items are: “Did you need help from nurses or other hospital staff in getting to the bathroom or in using a bedpan?” (Q10) leading to Q11; “Did you have any pain?” (Q12) leading to Q13 and Q14; “Were you given any medicine that you had not taken before?” (Q15) leading to Q16 and Q17; and “Did you go directly to your own home, to someone else’s home, or to another health facility?” (Q18) leading to Q19 and Q20. The HCAHPS questionnaire uses several different response scales: a dichotomous scale (1 = Yes, 2 = No), a global rating scale (0 = Worst to 10 = Best), and a four-point Likert scale (1 = Never, 2 = Sometimes, 3 = Usually and 4 = Always). The HCAHPS has a good factor loading value for all items, ranging from 0.70 to 0.81, and a good internal consistency reliability with a Cronbach’s alpha of 0.69 from its development process [19]. Questions 1–22 in the HCAPHS survey are in the public domain and therefore are not subject to United States copyright laws [19].

There were two phases involved in this study: Phase 1: Translation and adaptation of the original HCAHPS in English into Malay, Phase 2: Validation of the translated HCAHPS-Malay version in terms of content validation, face validation, and psychometric analysis.

### 2.2. Translation and Validation Process

In Phase 1, the original HCAHPS in English was translated into Malay, based on recommended guidelines for translation and cross-cultural adaptation [21,22]. For the forward translation process, two bilingual experts were used to translate the HCAHPS English version into Malay. A meeting to reconcile and review the HCAHPS-Malay version was conducted to ensure no change in the meaning of the content (content equivalence) from the translation process, and a consensus on the draft HCAHPS-Malay version was reached. Then, the backward translation was carried out by two additional bilingual experts, who translated the draft HCAHPS-Malay version back into English. A meeting to reconcile the translated and original English versions of the HCAHPS was held to determine the accuracy of the translation by identifying the differences between the two versions (semantic equivalence). In this reconciliation, both versions were compared for conceptual equivalence to ensure both versions were identical in context and meaning of the theoretical construct. All the translators in this study were proficient in both English and Malay.

In Phase 2, the HCAHPS-Malay version was validated for its content validity and face validity and further analyzed with psychometric analysis. Content validation entails assessment of the relevance and representativeness of each item to the specific domain by the expert panel. The HCAPHS-Malay version underwent content validation by a panel of 10 experts. The panel comprised three hospital management personnel, four members of the public, who were former patients with a history of admission, and three hospital management lecturers. The four members of the public were considered ’experts’, since they had experience with health services delivery during their hospitalization. The experts were asked to predict the relevance of the items related to the experience of hospital care in the country and population of interest by giving a score of 1 (item not relevant) to 4 (item very relevant), based on the relevance of the items in the translated HCAHPS.

The HCAHPS-Malay version then underwent pilot testing for face validation with 10 discharged patients selected by a purposive sampling method from one general medicine ward in Hospital USM. The inclusion and exclusion criteria used in this study were similar to the criteria from the original HCAHPS version: (1) patients able to understand the question; (2) patients without a cognitive disability; (3) patients above 18 years of age; and (4) discharged patients who are medically fit. A medically fit discharge patient was defined as needing care that can safely be continued in a nonacute setting such as at home or in a community setting [23]. The participants in the pilot test evaluated the overall features of the instrument and whether the sentences were clear and easy to understand, with appropriate layout and presentation. The face validity—the clarity and comprehension of the HCAHPS-Malay version—was assessed through a four-point Likert-scale response. For clarity, the scale ranged from a score of 1 (not clear at all) to 4 (very clear), and for comprehension from 1 (unable to understand at all) to 4 (easily understood). The feedback from the pilot testing was used for the correction and fine-tuning of the HCAHPS-Malay version by the research team.

The field testing of the HCAHPS-Malay version for psychometric analysis was conducted using different respondents from the pilot testing. This was a cross-sectional study conducted from January to February 2019. According to Hair Jr [24], with a subject-to-item ratio of 10:1, the minimum sample required in this study was 180, as the original HCAHPS consisted of 18 items. After taking into consideration a 10% non-responder rate, the sample size required for this study was 200. Simple random sampling was used to select the 200 participants from 13 wards, with at least 15 from every selected ward. Thirteen were selected randomly, based on three disciplines out of a total of 39 wards. They comprised five wards from the medical discipline, five wards under the surgical discipline, and three wards under the obstetrics and gynecology discipline. The general medical, cardiology, and nephrology wards were considered as the medical discipline; the general surgery, orthopedic, ophthalmology, neurosurgical, and otolaryngology wards were categorized as surgical; and the antenatal–postnatal and gynecology wards were categorized as the obstetrics and gynecology discipline. The selected patients were approached and invited to participate in the study. Those who agreed were screened for eligibility according to the study criteria, which were the same as the original HCAHPS version, as described above. The respondents were given the self-administered HCAHPS-Malay questionnaire to answer.

### 2.3. Statistical Analysis

Data entry and statistical analysis were performed using Microsoft Excel and IBM SPSS Statistics (Version 22, Armonk, NY, IBM Corp, Statistical Package for the Social Sciences, USA). For the descriptive analysis, categorical variables were presented as frequency and percentages. The normally distributed continuous variables were presented as mean and standard deviation (SD).

#### 2.3.1. Content Validity and Face Validity

The content validity index (CVI) and face validity index (FVI) were computed using Microsoft Excel. For the CVI analysis, the score from the expert panel was recategorized as 1 for relevant (scores 3 and 4) and as 0 for not relevant (scores 1 and 2). The CVI can be in two forms: (1) i-CVI: an average of all raters’ evaluations of an item, and (2) s-CVI: a scale level [25]. For FVI analysis, the score from 10 samples in the pilot study was recategorized as 1 for clear and understandable (scores 3 and 4) and as 0 for not clear and understandable (scores 1 and 2). The universal FVI was calculated by averaging the values of clarity and comprehension. The FVI was computed by calculating the scale average of the universal value [25].

#### 2.3.2. Construct Validity

The construct validity of HCAHPS-Malay was assessed by performing exploratory factor analysis (EFA) using principal axis factor analysis (PAF) with varimax rotation using IBM SPSS version 22. This analysis was conducted on the 200 data items from the field-testing cross-sectional study. The PAF was chosen, as we believed that there were latent variables underlying the item measure [26]. The underlying variables expected in this study were similar to the nine factors in the original HCAHPS, which are communication with nurse, communication with doctor, responsiveness of hospital staff, pain management, communication about medicine, hospital environment, discharge information, global rating, and hospital recommendation. Therefore, in this study, nine factors were requested. The Varimax rotation was chosen because none of the factors were correlated based on Tabachnick and Fiddell [27]. The orthogonal rotation that is the Varimax rotation can be chosen after the oblique rotation has been tested first and produced a correlation matrix of less than 0.32. The number of items to include in a factor or domain was determined by the factor loading greater than or equal to 0.4 [28].

#### 2.3.3. Internal Consistency

The internal consistency reliability of the HCAHPS-Malay version was analyzed using IBM SPSS, by determining the mean interitem correlation and Cronbach’s alpha. The mean interitem correlation was analyzed to determine the correlation among the items under the factor. A mean interitem correlation greater than 0.6 indicates that the item is highly correlated under its domain [24]. For the Cronbach’s alpha analysis for each domain, the single-item scales, such as the global rating that uses scale 1 to 10 and the item “recommendation to others”, could not be analyzed. Cronbach’s alpha values between 0.7 and 0.9 were considered to show high internal consistency [29].

### 2.4. Ethical Consideration

The HCAPHS questionnaire is in the public domain and does not require permission to use. This study obtained ethical approval from the Human Research Ethics Committee USM, Malaysia (USM/JEPeM/18100605). The data from this study have been strictly maintained for their confidentiality and will not be disclosed to any third party. Only the research team had access to the data. No personal information has been mentioned in the reporting and publication. Participation was on a voluntary basis and no remuneration was provided.

## 3. Results

### 3.1. Content Validity and Face Validity

#### 3.1.1. Content Validation

During the content validation process, all four screening questions, Q10, Q12, Q15, and Q18, were removed, based on the expert panel’s consensus. According to Khoie et al. [11], the screening questions do not provide any information on patient perception and can be removed from the questionnaire analysis. The content of Q11, “get help in getting to the bathroom or in using a bedpan”, was changed to “get help to take care of you”. The changes were made to suit the objectives of the study, local language, and culture. The overall CVI was calculated to be 0.87 and the iCVI for individual items ranged from 0.8 to 1.0 (Table 1).

#### 3.1.2. Face Validation

Table 1 shows the FVI results. For clarity, the FVI was 0.83 and for comprehension it was 0.82; the universal FVI was 0.82, indicating a satisfactory level of face validity. For the communication with nurse factor (item: 1, 2, 3), the iFVI (universal) range was between 0.8 and 0.85, and a little higher for communication with doctor (item: 5, 6, 7) at between 0.85 and 0.9. For the hospital staff responsiveness factor, the iFVI (universal) ranged between 0.75 and 0.8 and between 0.8 and 0.85 for the hospital environment factor. The iFVI (universal) for pain management ranged between 0.75 and 0.8 and it was 0.85 for the communication about medicine factor. For discharge information, the iFVI (universal) ranged between 0.7 and 0.75. For the global rating factor, the range was between 0.8 and 1.0.

### 3.2. Socio-Demographic Characteristics

For the construct validity and internal reliability testing, the field study was conducted on 200 patients discharged from wards in Hospital USM. The respondents’ socio-demographic characteristics are shown in Table 2. The distribution of sex among the respondents was almost equal. Of the respondents, 79.5% were less than 60 years of age (with a mean age of 44.9), and most respondents were married (73%). Almost all respondents had some formal education, but only a quarter of them claimed to have tertiary education. Nearly half (48%) of the respondents were from the medical ward, and the rest were from surgical-based and the obstetric and gynecology wards.

### 3.3. Exploratory Factor Analysis (EFA)

For construct validity, the Kaiser–Meyer–Olkin test showed sample adequacy with a value of 0.757, which exceeded the minimum requirement of 0.7 [30]. In addition, the Bartlett’s test showed significant results indicating the items were correlated and able to proceed for factor analysis. All the items in nine domains had a factor loading >0.4; therefore, no item was removed. The nine domains accounted for 83.49% of the total variance. The variance percentage explained by the individual domains 1, 2, 3, 4, 5, 6, 7, 8, and 9 after rotation were 13.63%, 10.91%, 10.69%, 10.45%, 9.19%, 8.94%, 8.75%, 5.55%, and 5.37%, respectively. The final HCAHPS-Malay version consists of 18 items within 9 domains. Table 3 shows the result of the items and factor loadings for the rotated factors.

Table 4 shows the results of the internal consistency reliability analysis. The Cronbach’s alpha for each domain ranged from 0.689 to 0.927. A Cronbach’s alpha value of more than 0.70 is considered as satisfactory internal reliability [29], and the higher alpha value indicates a higher internal reliability. In this study, the mean interitem correlation was also analyzed and ranged from 0.536 to 0.864. A mean interitem correlation more than 0.6 indicates that the item highly correlated under its domain [24].

## 4. Discussion

Patient experience of care assessment has been increasingly used to evaluate the service quality of the healthcare system. One of the instruments to assess it is the HCAPHS questionnaire, which is a standardized tool used since 2006 to measure patient experience in the USA. The HCAHPS questionnaire has been translated into many languages in order to be used in other countries, so that comparisons of health services quality can be made. Studies from other countries have found that the HCAHPS is relevant for patient care experiences assessment in these countries [13,15]. This study aimed to translate and validate the HCAHPS into the Malay language and is the first report of the translation and validation of the HCAHPS questionnaire in Malaysia.

The translated HCAHPS-Malay version provided valid and reliable results when it was applied. The potential threat to validity related to language translation is reduced significantly based on the findings of this study. The content equivalence and semantic equivalence were checked in this study to ensure that the quality and consistency of the meaning in the translated version were consistent with the original version. The translation and adaption processes in this study conformed to those used in previous studies related to HCAHPS [15,21,22]. Squires et al. [21] recommend that the instruments that are translated need to undergo content, context, conceptual, semantic, and technical equivalence evaluation to ensure the instrument is appropriate for use in the new setting. Failure to assess these components will lead to significant issues related to contextual and conceptual equivalence [21].

In this study, the iCVI scale obtained a score of 0.87, which is considered excellent, indicating that the content of the translated HCAHPS-Malay version is well adapted into the local context. A CVI score of above 0.8 indicates that the items in the questionnaire are relevant to the domain [25]. By comparison, a study on HCAHPS translation into the German and Italian languages in Switzerland obtained iCVI scores of 0.68 and 0.63, respectively [15]. In the same study, however, the iCVI for the translated HCAHPS in the Dutch, French, Finnish, Greek, and Polish languages ranged from 0.82 to 0.99. Based on our study, combining expertise from researchers and members of the public who had experienced health services delivery has a better evaluation of the relevance of the patient perception question. The FVI results in this study showed that the original HCAHPS was translated well into the Malay language using clear and understandable sentences. The satisfactory level of the FVI was taken at 0.8 and above, adopted from the CVI value [25].

The psychometric properties of the translated HCAHPS-Malay version were also analyzed. Previous studies found that the construct validity of HCAHPS was inconclusive in terms of the number of factors and its factor loading value. During the development process of the original HCAHPS, nine factors were extracted with a good factor loading value for all items ranging from 0.70 to 0.81 [20]. However, a study by O’Malley, Zaslavsky [31] determined only six factors extracted. Keller et al. [32] determined 7 factors consisting of 16 scale items. The latest study by Westbrook et al. [33] that used EFA determined 3 factors loaded with 18 scale items.

In our study, the EFA using PAF was done by fixing the number of extracted factors at nine to match with the original HCAHPS. This analysis is considered as EFA even though there was some idea about the structure of the item based on the original HCAHPS [26]. This is because the hypotheses regarding the model were not very specific in terms of prediction of the size of the observed variables related to each latent variable (domain). Moreover, the factor analysis was allowed to find factors that best fit into the data. Findings from this analysis showed that the translated HCAHPS-Malay version had a good factor loading of more than 0.4 under nine factors, and this is consistent with the initial validation of the original HCAPHS questionnaire [19]. The good result in this analysis was due to the rigorous methods of translation and cross-adaptation by content validation and face validation.

The HCAHPS-Malay version’s Cronbach’s alpha for patient experience factors ranged between 0.69 and 0.93, which exceeds the minimum value of 0.70 for internal consistency [29]. This is consistent with reliability values from previous studies on translation of HCAHPS into non-English languages, including Dutch, French, German, Greek, Italian, and Spanish [13,15]. The study by Kemp et al. [10] also showed a high Cronbach’s alpha of 0.90 overall and a range from 0.70 to 0.96 for individual domains, which indicated that the items were correlated under their domain. The mean interitem correlation for each factor ranged between 0.54 and 0.86, which exceeded the minimum 0.6 to indicate that the items under each factor were correlated [24]. The mean interitem correlation is more appropriate for the domains with only two or three items [24]. The findings in this study further confirm the original HCAHPS validation process in which the developers kept the number of items in each scale small (2–3 items) and the number of response categories for scale items small (e.g., 4-point scales) due to practical considerations, so that any patient with any level of education could comprehend and respond easily [33].

### Study Limitations

The results from our study may not be representative for Malaysia, since almost all the respondents in this study were from the Malay ethnic group, which limits its generalizability. Moreover, during the translation process, there were linguistic and administrative problems. For example, the inconsistencies in the translation of certain English affixes into Malay resulted in different words that carry the same concepts. In addition, selection bias of the expert panel may have occurred, especially with the selection of patients as expert panelists. Clear guidelines for selection of the raters need to be developed, since some of the raters had difficulty in evaluating questions instead of answering them.

## 5. Conclusions

The translated HCAHPS-Malay version has good construct validity and excellent absolute reliability in addition to content validity and face validity. Therefore, the translated HCAHPS-Malay version is a valid and reliable tool to determine patient perception, especially to reflect inpatient hospital services in Hospital USM, Malaysia. It is recommended that the patient perception survey using the HCAHPS-Malay versions is conducted in different population and healthcare settings in Malaysia for cross-validation studies. Thus, the HCAHPS-Malay version can become one of the valid indicators to measure healthcare system quality, leading to the improvement of healthcare services in local hospitals and the Malaysian healthcare system in general.

## Figures and Tables

**Table 1:** Content validity and face validity of the HCAHPS-Malay version questionnaire.

	iCVI	iFVI		
		Clarity	Comprehension	Universal
Communication with Nurse *Komunikasi dengan Jururawat* Q1: During this hospital stay, how often did nurses treat you with courtesy and respect? (1-Never, 2-Sometimes, 3-Usually, 4-Always) *Sepanjang berada di hospital ini, berapa kerap jururawat melayan anda dengan belas ihsan dan hormat?* *(1-Tidak Pernah, 2-Kadang-Kadang, 3-Selalu, 4-Sentiasa)*	1.0	0.8	0.8	0.8
Q2: During this hospital stay, how often did nurses listen carefully to you? (1-Never, 2-Sometimes, 3-Usually, 4-Always) *Sepanjang berada di hospital ini, berapa kerap jururawat mendengar dengan teliti kepada anda?* *(1-Tidak Pernah, 2-Kadang-Kadang, 3-Selalu, 4-Sentiasa)*	0.8	0.9	0.8	0.85
Q3: During this hospital stay, how often did nurses explain things in a way you could understand? (1-Never, 2-Sometimes, 3-Usually, 4-Always) *Sepanjang berada di hospital ini, berapa kerap jururawat menerangkan sesuatu perkara dengan cara yang boleh anda fahami?* *(1-Tidak Pernah, 2-Kadang-Kadang, 3-Selalu, 4-Sentiasa)*	0.9	0.8	0.8	0.8
Communication with Doctor *Komunikasi dengan Doktor* Q5: During this hospital stay, how often did doctors treat you with courtesy and respect? (1-Never, 2-Sometimes, 3-Usually, 4-Always) *Sepanjang berada di hospital ini, berapa kerap doktor melayan anda dengan belas ihsan dan hormat?* *(1-Tidak Pernah, 2-Kadang-Kadang, 3-Selalu, 4-Sentiasa)*	0.9	0.8	0.9	0.85
Q6: During this hospital stay, how often did doctors listen carefully to you? (1-Never, 2-Sometimes, 3-Usually, 4-Always) *Sepanjang berada di hospital ini, berapa kerap doktor mendengar dengan teliti kepada anda?* *(1-Tidak Pernah, 2-Kadang-Kadang, 3-Selalu, 4-Sentiasa)*	0.8	0.9	0.9	0.9
Hospital Staff Responsiveness *Tindakbalas staf hospital* Q4: During this hospital stay, after you pressed the call button, how often did you get help as soon as you wanted it? (1-Never, 2-Sometimes, 3-Usually, 4-Always) *Sepanjang berada di hospital ini, selepas memanggil jururawat, berapa kerap anda mendapat bantuan secepat yang anda inginkan?* *(1-Tidak Pernah, 2-Kadang-Kadang, 3-Selalu, 4-Sentiasa)*	0.9	0.8	0.8	0.8
Q10: How often did you get help in getting to the bathroom or in using a bedpan as soon as you wanted? (1-Never, 2-Sometimes, 3-Usually, 4-Always) *Berapa kerap anda menerima bantuan daripada staf hospital untuk menguruskan diri anda sebaik sahaja anda memerlukannya?* *(1-Tidak Pernah, 2-Kadang-Kadang, 3-Selalu, 4-Sentiasa)*	0.8	0.7	0.8	0.75
Hospital Environment *Persekitaran Hospital* Q8: During this hospital stay, how often were your room and bathroom kept clean? (1-Never, 2-Sometimes, 3-Usually, 4-Always) *Sepanjang berada di hospital ini, berapa kerap wad anda dan bilik air dijaga dengan bersih?* *(1-Tidak Pernah, 2-Kadang-Kadang, 3-Selalu, 4-Sentiasa)*	0.8	0.8	0.8	0.8
Q9: During this hospital stay, how often was the area around your room quiet at night? (1-Never, 2-Sometimes, 3-Usually, 4-Always) *Sepanjang berada di hospital ini, berapa kerap kawasan di sekeliling wad anda berada dalam keadaan senyap di waktu malam?* *(1-Tidak Pernah, 2-Kadang-Kadang, 3-Selalu, 4-Sentiasa)*	0.9	0.9	0.8	0.85
Pain Management *Pengurusan Kesakitan* Q11: During this hospital stay, how often did hospital staff talk with you about how much pain you had? (1-Never, 2-Sometimes, 3-Usually, 4-Always) *Sepanjang berada di hospital ini, berapa kerap staf hospital bercakap dengan anda tentang tahap kesakitan yang anda alami?* *(1-Tidak Pernah, 2-Kadang-Kadang, 3-Selalu, 4-Sentiasa)*	0.8	0.8	0.8	0.8
Q14: Before giving you any new medicine, how often did hospital staff describe possible side effects in a way you could understand? (1-Never, 2-Sometimes, 3-Usually, 4-Always) *Sebelum anda diberikan ubatan yang baru, berapa kerap staf hospital menerangkan kesan sampingan yang mungkin berlaku dengan cara yang boleh anda fahami?* *(1-Tidak Pernah, 2-Kadang-Kadang, 3-Selalu, 4-Sentiasa)*	0.8	0.9	0.8	0.85
Discharge Information *Maklumat Berkaitan Kebenaran Pulang* Q15: During this hospital stay, did doctors, nurses, or other hospital staff talk with you about whether you would have the help you needed when you left the hospital? (1-Yes, 2-No) *Sepanjang berada di hospital ini, adakah doktor, jururawat atau staf hospital yang lain ada bercakap dengan anda sama ada anda boleh mendapat bantuan yang diperlukan apabila anda meninggalkan hospital?* *(1-Ya, 2-Tidak)*	0.9	0.7	0.7	0.7
Q16: During this hospital stay, did you get information in writing about what symptoms or health problems to look out for after you left the hospital? (1-Yes, 2-No) *Sepanjang berada di hospital ini, adakah anda mendapat maklumat bertulis tentang apakah gejala atau masalah kesihatan yang perlu diberi perhatian apabila anda meninggalkan dari hospital?* *(1-Ya, 2-Tidak)*	0.8	0.8	0.7	0.75
Global Rating *Penarafan Global* Q17: Using any number from 0 to 10, where 0 is the worst hospital possible and 10 is the best hospital possible, what number would you use to rate this hospital during your stay? (0-Worst hospital till 10-Best hospital) *Dengan menggunakan skala 0 sehingga 10, di mana 0 adalah kemungkinan hospital paling teruk dan 10 adalah kemungkinan hospital terbaik, apakah nombor anda akan berikan untuk menilai hospital ini sepanjang anda berada di sini?* *(0-paling teruk hingga 10-hospital terbaik)*	1.0	0.8	0.8	0.8
Q18: Would you recommend this hospital to your friends and family? (1-Definitely no, 2-Probably no, 3-Probably yes, 4-Definitely yes)) *Adakah anda akan mengesyorkan hospital ini kepada rakan-rakan dan keluarga anda?* *(1-Sudah pasti tidak, 2-Mungkin tidak, 3: Mungkin Ya, 4-Sudah pasti Ya)*	1.0	1.0	1.0	1.0
Overall	0.87	0.83	0.82	0.82

Note: iCVI: item level Content Validity Index, iFVI: item level Face Validity Index.

**Table 2:** Socio-demographic characteristic of the respondents (*n* = 200).

Variable	*n* (%)	Mean (SD)
**Age**		44.9 (17.1)
Less than 60 years old	159 (79.5%)	
60 years old or more	41 (20.5%)	
**Sex**		
Male	101 (50.5%)	
Female	99 (49.5%)	
**Race**		
Malay	161 (95.5%)	
Other	9 (4.5%)	
**Marital Status**		
Married	146 (73.0%)	
Single	41 (20.5%)	
Divorced	13 (6.5%)	
**Educational Status**		
No formal education	12 (6.0%)	
Primary school	19 (9.5%)	
Secondary school	114 (57.0%)	
Diploma/Degree	55 (27.5%)	
**Occupation**		
Employed by government	46 (23.0%)	
Employed by private sector or self-employed	78 (39.0%)	
Not working	76 (38.0%)	
**Ward Discipline**		
Medical	96 (48.0)	
Surgical	82 (41.0)	
Obstetrics and Gynecology	22 (11.0)	

**Table 3:** Exploratory factor analysis on the HCAHPS-Malay version questionnaire.

Items	Domain									**Communality**
	1	2	3	4	5	6	7	8	9	
Nurse treats with courtesy and respect	0.796									
Nurse listens carefully	0.707									0.760
Understandable nurse explanation	0.652									0.664
Doctor treats with courtesy and respect		0.892								0.722
Doctor listens carefully		0.802								0.859
Understandable doctor’s explanation		0.762								0.772
Nurse responsiveness			0.909							0.751
Other hospital staff responsiveness			0.896							0.834
Cleanliness of environment				0.87						0.822
Quietness of environment				0.842						0.928
Staff discuss level of pain					0.873					0.929
Staff discuss how to treat pain					0.834					0.820
Talk about the purpose of medication						0.864				0.793
Talk about side effects of medication						0.849				0.797
Staff offer help for discharge							0.934			0.795
Written discharge information							0.929			0.906
Hospital rating								0.961		0.982
Hospital recommendation									0.945	0.902
Eigenvalues	2.454	1.964	1.925	1.881	1.654	1.61	1.575	0.999	0.967	0.993
% of variance	13.63	10.91	10.69	10.45	9.19	8.94	8.75	5.55	5.37	

**Table 4:** Internal consistency reliability of the HCAHPS-Malay version questionnaire.

Patient Experience Dimension	Number of Items	Cronbach’s Alpha	Mean Interitem Correlation
Communication with nurse	3	0.769	0.528
Communication with doctor	3	0.857	0.790
Responsiveness of hospital staff	2	0.927	0.864
Pain management	2	0.740	0.588
Communication about medicine	2	0.689	0.536
Hospital environment	2	0.754	0.606
Discharge information	2	0.890	0.805
